# The Fat from Frozen Mammals Reveals Sources of Essential Fatty Acids Suitable for Palaeolithic and Neolithic Humans

**DOI:** 10.1371/journal.pone.0084480

**Published:** 2014-01-08

**Authors:** José L. Guil-Guerrero, Alexei Tikhonov, Ignacio Rodríguez-García, Albert Protopopov, Semyon Grigoriev, Rebeca P. Ramos-Bueno

**Affiliations:** 1 Food Technology Division, CeiA3, University of Almería, Almería, Spain; 2 Zoological Institute, Russian Academy of Sciences, Saint-Petersburg, Russian Federation; 3 Organic Chemistry Division, CeiA3, University of Almería, Almería, Spain; 4 Department of Mammoth Faunal Studies, Sakha (Yakutia) Republic Academy of Sciences, Yakutsk, Russian Federation; 5 Yakutsk Scientific Research Institute of Applied Ecology of the North, North-Eastern Federal University, Yakutsk, Russian Federation; Max Delbrueck Center for Molecular Medicine, Germany

## Abstract

The elucidation of the sources of *n*-3 fatty acids available for the humans in the Upper Palaeolithic and Neolithic is highly relevant in order to ascertain the availability of such nutrients in that time frame as well as to draw useful conclusions about healthy dietary habits for present-day humans. To this end, we have analysed fat from several frozen mammals found in the permafrost of Siberia (Russia). A total of 6 specimens were included in this study: 2 mammoths, i.e. baby female calf called “Lyuba” and a juvenile female called “Yuka”, both specimens approximately from the same time, i.e. Karginian Interstadial (41,000 and 34,000 years BP); two adult horses from the middle Holocene (4,600 and 4,400 years BP); and two bison very close to the Early Holocene (8,200 and 9,300 years BP). All samples were analysed by gas-liquid chromatography-mass spectrometry (GLC-MS) and GLC-flame ionization detector (GLC-FID). As demonstrated in this work, the fat of single-stomached mammals often consumed by Palaeolithic/Neolithic hunters contained suitable amounts of *n*-3 and *n*-6 fatty acids, possibly in quantities sufficient to meet the today's recommended daily intake for good health. Moreover, the results also suggest that mammoths and horses at that time were hibernators.

## Introduction

The intake of *n*-3 (omega-3) fatty acids (FAs) during the Palaeolithic has been recently studied in terms of the *n*-6:*n*-3 ratio in the human diet, with an increase in this ratio being observed in the present day. This fact is of particular relevance, because an understanding of ancestral human experience over evolution may provide key knowledge on human dietary needs to maintain and improve health and avoid chronic illnesses [1], [2]. Moreover, the sources of *n*-3 available to Palaeolithic humans have been subject to controversy [3]; thus the elucidation of the sources of *n*-3 FAs that may have fed Palaeolithic humans in critical evolutionary stages can help clarify human evolution.

With its low plant-animal subsistence ratio for northern hunter-gatherers, the Palaeolithic diet was probably dominated by animal foods, especially during the cold season when plant resources decline [4]. Among Palaeolithic animals, woolly mammoths (*Mammuthus primigenius* Blumenbach, 1799), were good options for human consumption. Mammoths were monogastric herbivores, having a digestive physiology and diet similar to that of the woolly rhinoceros and the horse [5]. Although the consumption of mammoths by Palaeolithic humans has been controversial in comparison with mass hunting of species such as horses and bison, several authors have found evidence of this dietary behaviour, as has been deduced from Valea-Morilor excavations [6]. Probably, the hunting of mammoths was possible by tracking such pachyderms; Palaeolithic hunters could have caused stampedes as a hunting strategy, and attacked animals that could not maintain keep up with the herd. Mainly calves and some older individuals could have been killed in this way, thereby providing significant amounts of fat to ancestral hunters [7]. Moreover, through combined climate and population models, it has been shown that mammoth hunting pressure was clearly involved in the extinction of this pachyderm [8], and evidence of hunting with stone weapons during the Palaeolithic has been found [9], [10], [11]. The prey or scavenged products of mammoths would have been dragged or carried to the caves where Palaeolithic humans lived, as shown in the cave of Spy, which yields almost 10,000 remains from Ice Age mammals, the most frequent species being horse, cave hyena, and woolly mammoth [7].

Some of the present authors have previously described the presence of thick layers of subcutaneous fat and even humps on the neck in mummified carcasses of the mammoths found in the permafrost of Siberia (Russia) [12], [13]. The use of the mammoth fat, which is a large organ rich in energy, could have provided substantial benefits to ancestral hunters. That is, one medium-sized mammoth could have nourished a group of 50 humans (either Cro-Magnon or Neanderthal) for at least 3 months [14], while at the low temperatures in which they lived, would facilitate the conservation of the carcasses. Furthermore, by eating the fat of mammoths, Palaeolithic humans could have obtained clean, low-protein energy for several days.

This paper reports on the FA profiles of the fat of some animals from the Ice Age to the Neolithic, discussing the possibility of fat use as an *n*-3 source for such hunters. Also, the possibility that some of these mammals were hibernating is discussed, as suggested by some of the FAs found.

## Materials and Methods

### Samples

Permission was received to examine the relevant specimens from museum collections. Samples from the frozen mummies were donated by the Salekhard Museum, the Museum of the Mammoth of North-Eastern Federal University, and the Academy of Sciences of the Yakutia Republic in Yakutsk (Russia). A total of 6 specimens were included in this study (Table 1): 2 mammoths, i.e. a female calf called “Lyuba” from Yamal Peninsula (north-western Siberia) and a young female called “Yuka” from the banks of Laptev strait (north-eastern Siberia), both specimens approximately from the same time period (Karginian Interstadial, 41,000 and 34,000 years BP, respectively). Two adult horses from Yakutia, one (horse Yukagir) from the same place as Yuka and the other one (horse Batagay) from the locality near settlement Batagay in the middle stream of the Yana River. Both carcasses were surprisingly of the same approximate age, from the middle Holocene (4,600 and 4,400 years BP). Two bison, i.e. a baby bison from Batagay (bison “Batagay”) and a complete body of an adult male from the same region as Yuka and the Yukagir horse (bison Yukagir), were both again very close in time, from the Early Holocene (8,200 and 9,300 years BP).

**Table 1:** Sample characteristics of frozen mammals.

Sample code	Animal	Organ	Years BP
MY	Juvenile mammoth “Yuka”	Fat from left hind leg	34,300
ML1	Baby mammoth “Lyuba”	Fat from intestines	41,000
ML2	Baby mammoth “Lyuba”	Fat from hump	41,000
ML3	Baby mammoth “Lyuba”	Fat from abdominal wall	41,000
ML4	Baby mammoth “Lyuba”	Fat under the skin of belly	41,000
HY	Horse “Yukagir”	Fat from hind leg	4,600
HB	Horse “Batagay”	Fat from hind leg	4,400
BY	Bison “Yukagir”	Fat under the skin on the belly	9,300
BB	Baby bison “Batagay”	Fat under the skin on the belly	8,200

The dried and conserved baby mammoth Lyuba is currently on display at the Salekhard Museum, while the other individuals are kept in the freezers of Museum of the Mammoth of North-Eastern Federal University and Academy of Sciences of Yakutia Republic in Yakutsk. The samples were kept in the freezers of Zoological Institute Russian Academy of Sciences in Saint Petersburg. Sterile conditions were maintained during the dissection procedures and using a special drill on the frozen carcasses. During the dissection, fat from Lyuba was taken from four different positions, including the hump on the neck. The samples from the other specimens were taken from the layers under the skin in the best preserved areas (Table 1).

### Oil extraction and transesterification

Simultaneous oil extraction and transesterification was done according to previous works [15]. From each sample, 50 mg were weighed in test tubes and n-hexane (1 mL) was added to each one. FA methyl esters (FAMEs) were obtained after adding 1 mL of the methylation mixture, which was composed by methanol:acetyl chloride (20∶1 v/v), and then heated at 100°C for 10 min. After cooling at room temperature, 1 mL of distilled water was added in each tube, after which the tubes were centrifuged at 3500 rpm for 5 min. The Upper hexane layer was removed for gas-liquid chromatography (GLC) analyses.

### GLC Analyses

Firstly, FAMEs were analysed by using a Focus GLC (Thermo Electron, Cambridge, UK) equipped with Flame Injection Detector (FID) and a Omegawax 250 capillary column (30 m×0.25 mm i.d. ×0.25 µm film thickness; Supelco, Bellefonte, PA, USA). The temperature programme was: 1 min at 90°C, heating until 200°C at a rate of 10°C/min, constant temperature at 200°C (3 min), heating until 260°C at a rate of 6°C/min and constant temperature at 260°C (5 min). The injector temperature was 250°C with a split ratio 50∶1. The injection volume was 4 µL and the detector temperature was 260°C. Nitrogen was used as the carrier gas (1 mL/min) and peaks were identified by retention times determined for known FAME standards (PUFAs No. 1 from Sigma, St. Louis, USA), while FA contents were estimated by using methyl pentadecanoate (17∶0) as internal standard.

All samples were subjected to a second round of analyses by GLC-mass spectrometry (GLC-MS) at the Scientific Instrumentation Centre of the University of Granada (Spain). Samples were injected (2 µl) into an Agilent 7890A gas chromatographer with an apolar column in split mode, coupled with a Quattro micro GLC mass spectrophotometer (Waters, UK), with a positive electron impact source (70 eV) and full scan spectra acquisition. All FAs were detected and quantified by comparison of retention times and mass spectra with external standards, which were run at three different concentrations. The full dataset of the GLC-MS analysis is available upon request.

Experiments for all samples were conducted at least in triplicate. Results are expressed as mean value ± S.D in Tables 2 and 3.

**Table 2:** Total fatty acids and saturated fatty acids composition of the fat from frozen mammals a.

		FAs % of total FAs										
Samples	Total FAs g/100 g tissue	10∶0	11∶0	12∶0	13∶0	MA 14∶0	15∶0	PA 16∶0	17∶0	SA 18∶0	20∶0	22∶0
MY	0.6±0.1	-	-	-	-	7.1±0.4	-	39.1±1.5	0.2±0.0	14.3±1.1	-	-
ML1	21.5±0.7	0.5±0.1	0.1±0.0	2.2±0.2	0.2±0.0	8.8±0.3	1.5±0.1	75.3±2.1	0.9±0.2	4.0±0.3	-	0.02
ML2	23.6±0.9	0.5±0.0	0.1±0.0	2.1±0.1	0.1±0.0	7.4±0.6	1.3±0.2	75.4±1.5	0.8±0.1	2.9±0.3	-	-
ML3	28.5±1.2	0.5±0.1	0.1±0.1	2.0±0.3	0.2±0.0	8.6±0.6	1.5±0.2	74.8±1.6	0.7±0.2	2.6±0.2	-	-
ML4	18.5±0.8	0.4±0.1	0.1±0.0	2.5±0.2	0.2±0.1	9.9±0.5	2.0±0.1	70.6±2.2	1.0±0.1	4.4±0.3	0.2±0.1	0.02
HY	23.8±1.0	-	-	0.1±0.0	-	3.2±0.4	0.2±0.0	36.9±2.0	0.1±0.0	3.8±0.2	-	-
HB	1.0±0.1	-	-	-	-	4.6±0.3	0.2±0.1	48.7±3.0	-	7.1±0.5	-	0.02
BY	16.3±1.2	-	-	-	-	8.0±0.2	-	48.0±2.8	0.1±0.0	28.0±1.7	-	-
BB	15.7±0.9	-	-	0.4±0.1	-	5.7±0.3	0.6±0.0	48.7±3.2	0.2±0.0	37.4±2.0	1.1±0.1	0.06

^a^ Mean ± SD of three independent determinations performed by GLC-MS.

**Table 3:** Unsaturated fatty acids composition of the fat from frozen mammals^a^.

	FAs % of total FAs																
				OA 18∶1													
Samples	14∶1	POA 16∶1	17∶1	*n-9E*	*n-7*	*n-9Z*	Total 18∶1	LA 18∶2*n*-6	GOA 20∶1*n*-9	ALA 18∶3*n*-3	EDA 20∶2*n*-6	ERA 22∶1*n*-9	DHGLA 20∶3*n*-6	ETE 20∶3*n*-3	AA 20∶4*n*-6	EPA 20∶5*n*-3	NVA 24∶1*n*-9
MY	-	7.1±0.5	-	-	-	28.5±1.8	28.5±1.8	3.6±0.3	-	0.01	-	-	-	-	0.01	-	-
ML1	-	0.4±0.1	0.2±0.0	2.1±0.3	0.4±0.1	3.0±0.2	5.4±0.6	0.2±0.0	0.1±0.0	0.08	0.04	0.03	0.02	0.07	0.02	0.09	0.1±0.0
ML2	-	0.9±0.2	0.3±0.1	1.4±0.2	0.7±0.2	5.8±0.6	7.9±0.6	0.1±0.0	0.1±0.0	0.09	0.03	-	-	0.08	-	-	-
ML3	-	0.6±0.1	0.3±0.0	1.9±0.3	0.8±0.1	4.9±0.3	7.6±0.4	0.2±0.0	0.1±0.0	0.09	0.03	-	-	0.07	-	-	-
ML4	-	0.6±0.2	0.3±0.1	4.3±0.3	-	3.4±0.4	7.6±0.5	0.3±0.1	0.1±0.1	0.08	0.04	0.03	-	0.04	0.02	-	-
HY	0.2±0.1	7.1±0.5	0.4±0.0	-	1.5±0.2	40.1±0.5	41.6±2.4	1.8±0.2	0.3±0.0	4.2±0.2	-	-	-	0.01	-	0.01	-
HB	-	2.1±0.2	-	-	0.7±0.1	36.7±2.0	37.4±1.1	-	-	-	-	-	-	-	-	-	-
BY	-	-	-	-	-	16.0±1.3	16.0±0.9	-	0.1±0.1	0.9±0.1	-	-	-	0.2±0.1	-	0.02	-
BB	-	-	-	0.7±0.2	-	4.6±0.5	5.3±0.4	-	-	-	-	-	-	0.06	-	-	-

^a^ Mean ± SD of three independent determinations performed by GLC-MS.

## Results and Discussion

The animals sampled in this study are representative of those eaten by Upper Palaeolithic and Neolithic hunters [4], [7], [11], [14]. Two species, mammoths and horses, were monogastric, while bison was a ruminant; thus, differences were expected in the FA profiles of their fat, because monogastric animals can to a larger extent assimilate most FAs from the food they eat [16]. A detailed analysis of the content of intestinal samples from a one-month-old female Siberian mammoth calf Lyuba, revealed the presence of large amounts of herbaceous plants, e.g. species belonging to Cyperaceae, Asteraceae, Salicaceae, Chenopodiaceae, Poaceae [17]. Some of these plant species are known to be good sources of polyunsaturated FAs (PUFAs) belonging to the *n*-3 series [18]. Therefore, the subcutaneous fat of mammoths should include suitable concentrations of α-linolenic acid (ALA, 18∶3*n*-3), which is the major FA in leaf tissues, and other *n*-3 FAs.

**Figure 1:**
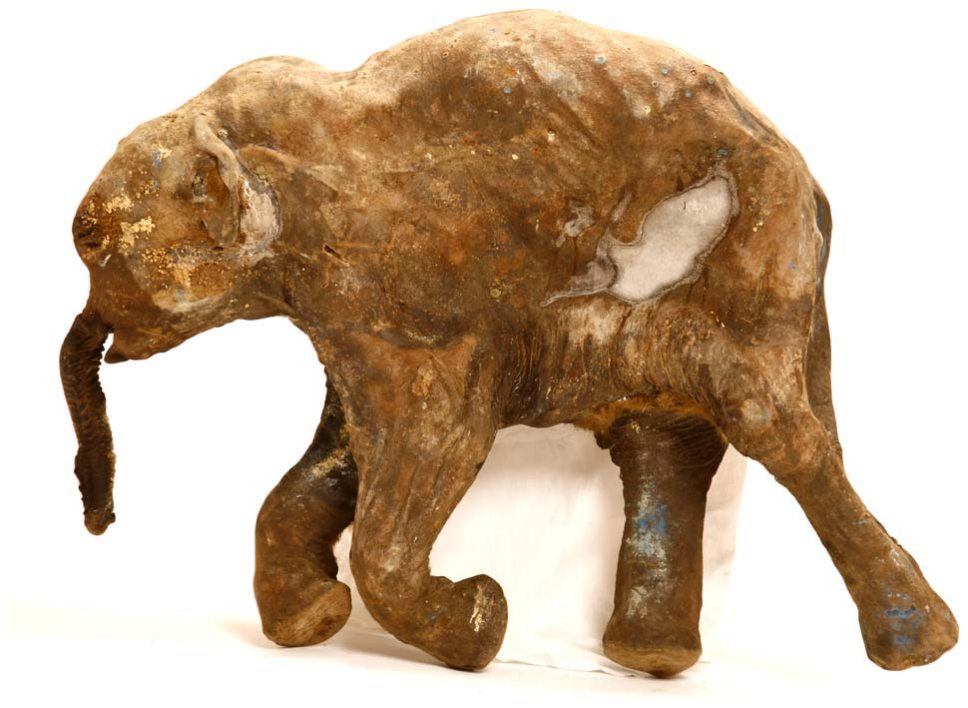
Mammoth calf Lyuba had a hump on the neck consisting of special fat cavities.

We hypothesised that the fat of monogastric animals constituted a raw source of *n*-3 and *n*-6 FAs for Upper Palaeolithic and Neolithic people. For this, we analysed the fat from various body parts of the mammoth Lyuba, and the juvenile female Yuka, two horses, and two bison (Table 1). Lyuba samples were collected from different areas in her body due to the discovery of the fat concentration (hump) on the neck of mammoth calves (Figure 1) [12], [13]. There is clear evidence that mammoths, even at a young age, stored large amounts of fat to survive during the long Arctic winter. Therefore, another purpose of this research was connected with the possibility of finding differences between the hump fat and the fat from other areas of the body. On the other hand, the fat from the most recent carcasses of extinct bison and horses (8,200–9,300 years BP and 4,400–4,600 years BP, respectively) in Siberia can provide information concerning similarities with modern Canadian forest bison and Yakutian horses.

**Figure 2:**
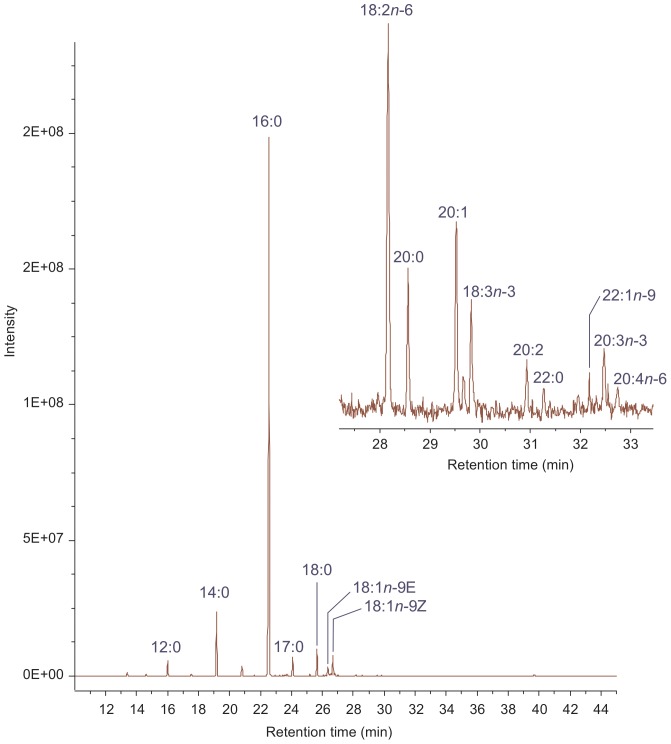
Gas-liquid chromatogram of fatty acid methyl esters from under skin fat from the belly of mammoth Lyuba. As noted in the chromatogram, palmitic acid (16∶0) is the main FA component, but the peaks due to the monounsaturated 18∶1*n*-9*E* and 18∶1*n*-9*Z* FAs can be clearly seen too.

**Figure 3:**
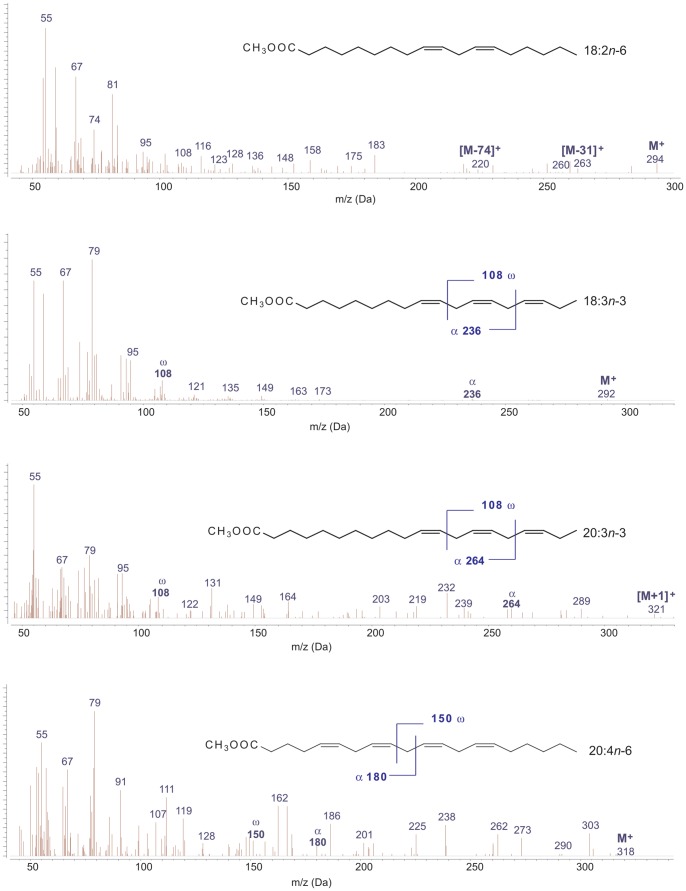
Selection of mass spectra of polyunsaturated fatty acids methyl esters. The typical ω fragmentation peaks at m/z 108 due to *n*-3 terminal groups, as well as the α fragments (at m/z 236 for 18∶3*n*-3, m/z 264 for 20∶3*n*-3, and m/z 180 for 20∶4*n*-6) are clearly seen. Note that the methyl group is a consequence of the derivatization, and was not present in the original sample.

A chromatogram of the FAs taken from under the belly skin of Lyuba (ML4) is plotted in Figure 2, while the mass spectra of a selection of PUFA showing their characteristic fragmentation patterns are displayed in Figure 3. The FA profiles shown here (Tables 2 and 3) are similar to others commonly found in the subcutaneous fat of present-days grass-feeding animals, such as elephant [19], horse [20], [21], and bison [22], although some differences in the FA percentages were found, as discussed below. On the other hand, hydroxylated FAs, which are associated with fat immersion in water, and that have previously been described in other frozen fats [23], were not detected here.

The subcutaneous fat of the baby mammoth Lyuba (ML1-ML4) contains noticeable amounts of saturated even-chain FAs: myristic acid (MA, 14∶0), palmitic acid (PA, 16∶0), and stearic acid (SA, 18∶0), and minor amounts of C11-17 saturated odd-FAs. In addition, quantifiable amounts of unsaturated even-FAs were present in all samples: palmitoleic acid (POA, 16∶1*n*-7), oleic acid (OA, 18∶1*n*-9), linoleic acid (LA, 18∶2*n*-6), gondoic acid (GOA, 20∶1*n*-9), ALA, eicosadienoic acid (EDA, 20∶2*n*-6), and eicosatrienoic acid (ETE, 20∶3*n*-3). Other FAs, such as erucic acid (ERA, 22∶1*n*-9), dihomo γ-linolenic acid (DHGLA, 20∶3*n*-6), arachidonic acid (AA, 20∶4*n*-6), eicosapentaenoic acid (EPA, 20∶5*n*-3), and nervonic acid (NVA, 24∶1*n*-9) were detected in some fatty tissues.

The juvenile mammoth Yuka (MY) contains only C17 as saturated odd-FA, high amounts of MA, PA and SA, and registered higher percentages of OA, LA, and POA than did the baby mammoth Lyuba, as well as minor amounts of ALA and AA. As in the mammoths, the two bison had different FA profiles: the one called bison Yukagir (BY) had a relatively high percentage of OA, while the one called bison Batagay (BB) lacked significant amounts of this FA, and registered higher amounts of saturated FAs than did bison Yukagir, both animals showing low amounts of PUFAs. Finally, both horses had high percentages of PA and OA, the one called horse Yukagir (HY) exhibiting several PUFAs: LA, ALA, ETE, and EPA.

The samples analysed differed in terms of FA preservation, although, given the high amounts of unsaturated FAs detected, the samples were generally better preserved than expected. However, following the above-mentioned analytical methodology, some other PUFAs were not detected, i.e., docosapentaenoic acid (DPA, 22∶5*n*-3), docosatetraenoic acid (DTA, 22∶4*n*-6) and docosahexaenoic acid (DHA, 22∶6*n*-3). This is a normal situation, considering the high degree of unsaturation of these latter PUFAs, which induces rapid degradation. In any case, we do not ruled out their appearance in other samples from frozen mammoths or horses having better preservation status than the present ones.

It was noticeable that most samples contained total FA amounts consistent with those that contemporary animals have in the same organs. However, two animals, the mammoth Yuka and the horse Batagay, yielded very low percentages. This could be due to the degradation of the samples and also to contamination of the original tissues by foreign substances such as hair and fur.

In mammoth tissues, besides OA and LA, ALA and ETE were found in almost all samples. The occurrence of these FAs in the plants that all these animals presumably ate is quite clear; these FAs also reach relatively high percentages in some lichen species characteristic of the Siberian tundra, such as *Cladina arbuscula* and *C. stellaris*, with concentrations above 1%, these species being consumed today by reindeer [24]. Other lichens that might have been consumed by mammoths, such as *Leptogium saturninum*, also contain concentrations of these PUFAs at the same level [25].

Lyuba shows an FA profile (samples ML1-ML4) that was probably influenced by milk intake, as it contained all the saturated C10-18 FAs, similar to those present in the milk of the current Indian elephant [26]. Furthermore, it has been cited that the milk of such pachyderms contains detectable amounts of ALA and ETE, which probably came directly from the animal's diet [26]. Therefore, assuming that mammoths had a metabolism similar to those of their current relatives, we can deduce that the FAs found in the subcutaneous fat of Lyuba came from mammoth milk, which would contain the FAs indicated above due to the mother's intake of lichens, mosses, and several plants.

It is well established that single-stomached animals, such as elephants, rhinoceroses and horses, are susceptible to changes in the FA composition in their adipose tissue as a result of the intake of fats having different FA profiles [15]. In fact, the carbon-carbon double bonds present in PUFAs are not hydrogenated during digestion and, hence, PUFAs can be incorporated into depot fat without modification [27], as recently established in grass-fed Yakutian and Galician horses [20], [21]. Thus, as in horses or elephants, the PUFA ingested by mammoths could have been incorporated into their body tissues, as in modern elephants, in which the PUFA content in the subcutaneous fat can reach nearly 25% of total FAs [19].

Although in this study all the FAs usually present in the depot fat of mammals have been detected, their relative proportions clearly differ from the original. It is widely accepted that frozen fats undergo a transformation in which the unsaturated FAs tend to disappear in favour of two units of shorter saturated FAs [28]. Thus, the FA profiles of the samples shown in Tables would be due to a combination of certain factors: the foods consumed by the animals, the physiology of their digestive system, and the extent of the FA transformation. By considering the FA profiles of the current relatives to the frozen animals studied here, the original FA profiles have been deduced (Table 4). The principle underlying this FA-profile reconstruction is that the amounts of PA and SA in the samples must be increased with the amounts coming from the transformed C18 PUFA (to PA) and C20 PUFA (to SA), as discussed above.

**Table 4:** Approximate original fatty acid profiles calculated for frozen mammals.

	FAs % of total FAs a,b					
	16:0 PA	18:0 EA	18:1*n*-9 OA	18:2*n*-6 LA	18:3*n*-3 ALA	C20 PUFA
MY c	*18* ^39.1^	*8* ^14.3^	28.5	*7* ^3.6^	*18* ^0.0^	*<6*
HY d	*18* ^36.9^	*2* ^3.8^	41.6	*7* ^1.8^	*18* ^4.2^	<*2*
HB d	*19* ^ 48.7^	*4* ^7.1^	37.4	*8* ^0.0^	*22* ^0.0^	*<3*
BY e	*32* ^48.0^	*27* ^28.0^	*29* ^16.0^	*2* ^0.0^	*1* ^0.0^	<*1*
BB e	*27* ^48.7^	*36* ^37.4^	*24* ^5.3^	*2* ^0.0^	*1* ^0.0^	<*1*

^a^ Only the major FAs have been considered.

^b^ A superscript number indicates the FA percentages found in frozen samples, while all derived percentages appear in italics. In reconstructed figures, C18 PUFA percentages are partially subtracted from PA figures; C20 PUFAs from EA. For both bison, reconstructed OA percentages are partially subtracted from PA.

^c^ PUFA ratios and PA percentage in agreement with those of grass-fed elephants [19].

^d^ PUFA ratios and PA percentage as grass-fed Siberian [20] and Galician horses [21].

^e^ PUFA ratios and PA percentage as grass-fed Bison subcutaneous fat [22].

In the case of the juvenile mammoth Yuka, the high amounts of OA found indicate that such a percentage is closely related to the original, whereas the PA percentage is clearly higher due to the transformation of pre-existent PUFAS —that is, LA and ALA became PA. Meanwhile, for SA this increase was due to the transformation of variable amounts of GOA, EDA, DHGLA, ETE, AA, and EPA. Other FAs encountered in high amounts in this mammoth were MA and POA, but in values similar to those usually found in hibernating animals such as badger, bear, and beaver [29]. In addition, as occurs in hibernating mammals, this mammoth shows minor amounts of several PUFAs, although the original PUFA percentages should be higher, as a large amount of these had to be transformed, as indicated above. Therefore, high percentages of LA and ALA should have been present in the original fat, which would have had strong effects on mammoth's hibernation [30]. This behaviour could have given an evolutionary advantage to mammoths, since the animals that hibernate have better chances of surviving the winter, when temperatures are low and food supplies are virtually nonexistent [30].

Such deductions concerning the possibility of mammoth hibernation are strongly supported by the behaviour of modern Yakutian horses; that is, during the winter and strong frosts, although they move somewhat, they stay mainly in sleeping position with minimal feeding. Also, this horse has an unusual thick layer of fat under the skin and in the abdominal cavity. Presumably, the woolly mammoth in the Arctic zone of Siberia showed a similar behaviour. In such areas, during the long and dark winter, the animals could semi-hibernate with minimal activity due to the presence of that special kind of fat in their tissues.

For the baby mammoth Lyuba, the original FA profile would be highly speculative to deduce, given both the animal's infant status and the high content of PA, which would come from either OA, LA, and/or ALA. On the other hand, the tissues of this animal contain ETE, a PUFA usually found in fish oil and in hibernating mammals [31]. The difference between the two specimens of mammoth may be related not only to their individual ages but also to the season of their death. Lyuba died at the beginning of summer (1–2 months old), but Yuka, as the wool covering with thick underwool suggests, died during the cold period (late autumn to winter).

To reconstruct the original FA profile of bison and horses, we undertook a process similar to that explained above (Table 4). In the case of horses, LA and ALA percentages were partially subtracted from PA ones, and distributed according to the proportions detected in the subcutaneous fat of other living horses related to those considered here [20], [21]; with the assumption that the current OA percentage is probably very close to the original. In the case of the bison, the ratios among FAs were taken from literature [22].

In addition to this reconstruction, a remarkable finding was made in the fat of horse Yukagir (HY), which is the high amount of ALA detected (4.2%). Such a quantity had never been detected in any sample from frozen prehistoric animals or humans; only in some cases have minor amounts of LA been discovered, as in the Tyrolean Iceman [23]. This unexpected finding suggests a high intake of ALA by these animals, leading to percentages of around 20% in their subcutaneous fat (Table 3), which could have contributed to fulfil the daily needs of *n*-3 FAs for hunters at the Neolithic, i.e. the time in which these animals lived. On the other hand, the reconstructed FA profiles of both horses are closely similar to that shown by current Yakutian horses [20], suggesting that this ancestor also semi-hibernated.

The FA profiles described here have clear implications for Stone Age humans. Although most long-chain PUFAs have been transformed, the remaining amounts indicate the ability of fatty tissues to store them. Given the high proportion of PUFAs deduced for these frozen animals, despite the difficulty of determining the exact quantity of fat in the unaltered carcasses, and under the assumption that mammoths would have required a large amount of subcutaneous fat in their bodies to survive in extremely cold environments, the mammoths could have satisfied the daily *n*-3 needs of Palaeolithic hunters. The same deduction could be proposed for horse, but in this case for humans at the middle Holocene, i.e. the age to which the analysed samples have been attributed. An added advantage of the use of this organ as a PUFA source might have been that the fat obtained from carcasses could have been preserved for long periods of time until consumption.

The results of this study indicate that the monogastric animals analysed, i.e. the woolly mammoth and the horse, might have had a hibernating or semi-hibernating behaviour, while their subcutaneous fat could have been consumed by Stone Age hunters to fulfil the daily needs in essential FAs.
